# TNF-Mediated Homeostatic Synaptic Plasticity: From *in vitro* to *in vivo* Models

**DOI:** 10.3389/fncel.2020.565841

**Published:** 2020-09-30

**Authors:** Renu Heir, David Stellwagen

**Affiliations:** Department of Neurology and Neurosurgery, Centre for Research in Neuroscience, Research Institute of the McGill University Health Center, Montréal, QC, Canada

**Keywords:** inflammation, homeostatic plasticity, addiction, developmental plasticity, TNF

## Abstract

Since it was first described almost 30 years ago, homeostatic synaptic plasticity (HSP) has been hypothesized to play a key role in maintaining neuronal circuit function in both developing and adult animals. While well characterized *in vitro*, determining the *in vivo* roles of this form of plasticity remains challenging. Since the discovery that the pro-inflammatory cytokine tumor necrosis factor-α (TNF-α) mediates some forms of HSP, it has been possible to probe some of the *in vivo* contribution of TNF-mediated HSP. Work from our lab and others has found roles for TNF-HSP in a variety of functions, including the developmental plasticity of sensory systems, models of drug addiction, and the response to psychiatric drugs.

## Homeostatic Synaptic Plasticity (HSP)

The maintenance of neural circuit function is a dynamic balance of several different types of synaptic plasticity. Synaptic strength can be modified by two broad types of plasticity mechanisms: Hebbian and non-Hebbian. Long term potentiation (LTP) and long term depression (LTD) are examples of Hebbian plasticity, where the strength of a given synapse is adjusted in response to synchronous activity (Malinow and Malenka, 2002). It is proposed as a mechanism of information storage and is thought to underlie the processes of learning and memory. On the other hand, non-Hebbian plasticity is posited to serve a homeostatic role, maintaining the stability of neural circuits in the face of changing conditions (Turrigiano et al., 1998).

Homeostatic synaptic plasticity (HSP) serves to keep neuronal activity levels in a range optimal for neurotransmission. It was first described as a response to prolonged perturbations in overall activity levels: when firing rates decrease, it serves to augment excitatory synaptic strength to normalize activity (sometimes referred to as upscaling), and when firing rates increase, the opposite occurs (downscaling). This phenomenon has been described in a variety of systems, including the mammalian central nervous system and the *Drosophila* neuromuscular junction (NMJ). The HSP at the *Drosophila* NMJ appears mechanistically distinct from HSP in the mammalian CNS, and therefore will not be covered here (for reviews of this topic see Davis and Müller, 2015; Frank et al., 2020). It should be noted, however, that both innate immune molecules and glia have recently been implicated in HSP at the *Drosophila* NMJ (Harris et al., 2015; Wang et al., 2020).

For the mammalian system, since being first described in the late nineties (O’Brien et al., 1998; Turrigiano et al., 1998), a great diversity of molecules have been implicated in homeostatic alterations in synaptic strength. These include proteins involved in calcium signaling (Thiagarajan et al., 2002; Ibata et al., 2008), transmembrane signaling proteins including MHCI and integrins (Goddard et al., 2007; Cingolani et al., 2008), endocytic proteins like Arc (Rial Verde et al., 2006; Shepherd et al., 2006), cytoskeletal proteins such as synaptopodin (Vlachos et al., 2013) and Homer1a (Hu et al., 2010), receptor-interacting proteins including PICK1 (Anggono et al., 2011), Narp (Chang et al., 2010), polo-like kinase 2 (PLK2; Seeburg et al., 2005), and dystroglycan (Pribiag et al., 2014), and secreted factors including BDNF (Rutherford et al., 1998), retinoic acid (Aoto et al., 2008; Chen and Napoli, 2008), and the pro-inflammatory cytokine tumor necrosis factor (TNF; Stellwagen and Malenka, 2006).

From these reports, it is clear that HSP is more diverse than originally described; it is not a single process, but rather many mechanisms operating either in conjunction or in parallel, responding to distinct circumstances. For example, there is evidence for cell-specific forms of HSP distinct from HSP induced by global activity suppression (Burrone et al., 2002). On a subcellular level, there are reports of homeostatic control of local dendritic regions and synapse-specific forms of HSP (Sutton et al., 2006; Kim and Tsien, 2008; Beique et al., 2011; Petrus et al., 2015; Barnes et al., 2017). Furthermore, multiple types of HSP have emerged operating at different spatial and temporal scales (Lee et al., 2014), and even the global form of HSP may still have distinct temporal components, with a more rapid retinoic acid-dependent form (Chen et al., 2014) and a slower, longer-lasting TNF-dependent form (Stellwagen and Malenka, 2006; Steinmetz and Turrigiano, 2010). It is important to note that the TNF-dependent and retinoic acid-dependent mechanisms of HSP only mediate upscaling, while a similarly varied but distinct set of molecules and mechanisms contribute to downscaling (Seeburg et al., 2008; Pribiag et al., 2014). Thus upscaling and downscaling are likely to be separate phenomena.

In addition to assuming HSP would have a single mechanism, early work also suggested that these changes occur in a multiplicative fashion: synaptic strength is adjusted by the same factor such that the relative differences in synapses are preserved (Turrigiano et al., 1998) and therefore the information stored in the synaptic weight difference would also be preserved (Turrigiano and Nelson, 2004). As a result, HSP is often referred to as synaptic scaling. This hypothesis may not strictly hold: recent reports that while the multiplicative nature of scaling holds true on a population level, there is variable scaling at the level of individual synapses (Wang et al., 2019; Hanes et al., 2020). Recent results have also challenged the notion that changes in cell firing are the driver for HSP, as maintaining spiking while blocking synaptic function still leads to HSP (Fong et al., 2015). Consequently, we shall avoid the term synaptic scaling, and only use HSP instead.

One of the first proteins placed within this pathway was TNF (Stellwagen and Malenka, 2006). This review article will first explore the effect of TNF on synapses, and then explore the models and systems in which TNF mediates different forms of homeostatic plasticity.

## TNF in the Brain

Historically, the central nervous system (CNS) was considered a site that was kept separate from the peripheral immune system, with immune signaling molecules excluded from the CNS by the blood-brain barrier (BBB; Barker and Billingham, 1977). The two systems were thought as so distinct that a specific term was coined to describe how they were kept separate: immune privilege. The lack of conventional lymphatic vessels as well as the extended survival of foreign tissue grafts in the brain suggested that the CNS is not capable of the same immune responses that are present in the periphery. The first evidence to the contrary was the discovery that under some pathological conditions, cytokines, mediators of immune responses, are produced in the brain (Hopkins and Rothwell, 1995). Furthermore, it is now becoming evident that immune privilege is far from absolute (Galea et al., 2007) and immune molecules are present in the nervous system even under non-pathological conditions and play a role in regulating synaptic function (Vitkovic et al., 2000). In particular, the pro-inflammatory cytokine TNF regulates synaptic properties and has been ascribed a role in HSP (Stellwagen and Malenka, 2006).

## TNF and TNF Receptor Overview

Cytokines are small protein molecules released by cells that serve as messengers between immune cells, modulating their interactions and behavior. TNF is one such pleiotropic cytokine that has many well-characterized roles including mediating inflammatory responses, cell differentiation, and organogenesis (Locksley et al., 2001; Santello and Volterra, 2012). It is transcribed as a single pass transmembrane pro-protein which can signal directly in its membrane-bound form (Grell et al., 1995). It can also be cleaved by the matrix metalloprotease ADAM17 (otherwise known as TNF- converting enzyme; TACE) to release soluble TNF (Kriegler et al., 1988; Black et al., 1997). Regardless of its cleavage status, TNF forms trimers which are the active form, responsible for signaling at TNF receptors (TNFRs; Smith and Baglioni, 1987).

TNF is produced in the CNS during a variety of inflammatory pathologies. It is upregulated after exposure to bacterial and viral proteins (Lokensgard et al., 2001; Kielian et al., 2002), but can also be induced by intrinsically-derived CNS insults. It is increased in diseases such as multiple sclerosis (MS; Hofman et al., 1989), Alzheimer’s disease (AD; Fillit et al., 1991), Parkinson’s disease (PD; Mogi et al., 1994), among others. Acute injuries such as CNS trauma also result in TNF expression (Ross et al., 1994). In addition to a role in the CNS in response to these various pathologies, both TNF mRNA and protein can be found in the non-inflamed brain (Vitkovic et al., 2000), suggesting functions even under non-pathological conditions.

The concentration of TNF is likely significant—low, physiological levels seem to modulate neuronal function, considerably below the high concentrations found in inflammatory or disease states. TNF levels are constitutively low and only modestly increase (3–5 fold) with activity blockade or other manipulations (Stellwagen and Malenka, 2006; Lewitus et al., 2016). This review article will address TNF at physiological, not pathological, concentrations.

TNF can signal through two receptors—TNFR1 and TNFR2—which differ in their expression pattern, binding affinity for the different forms of TNF, and their downstream signaling pathways (MacEwan, 2002). TNFR1 can efficiently bind both soluble and membrane-bound TNF while TNFR2 has a much higher affinity for binding to membrane-bound TNF (Grell et al., 1995). TNFR1 is constitutively expressed by cells in the CNS (Kinouchi et al., 1991) and periphery (Aggarwal, 2003), whereas expression of TNFR2 is more limited, with reports mainly in endothelial and immune cells (Aggarwal, 2003) as well as reports of expressions in some neurons (Neumann et al., 2002). TNFR1 signaling is complex, and can result in proliferation, activation, and apoptosis, depending on the context, while TNFR2 signaling is generally anti-inflammatory and pro-survival (Wajant et al., 2003). Additionally, membrane-bound TNF can signal in the reverse direction when complexed with TNFR1 in both the immune and nervous systems (Harashima et al., 2001; Kisiswa et al., 2013).

## TNF Effects on Pyramidal Neurons

**Figure 1:**
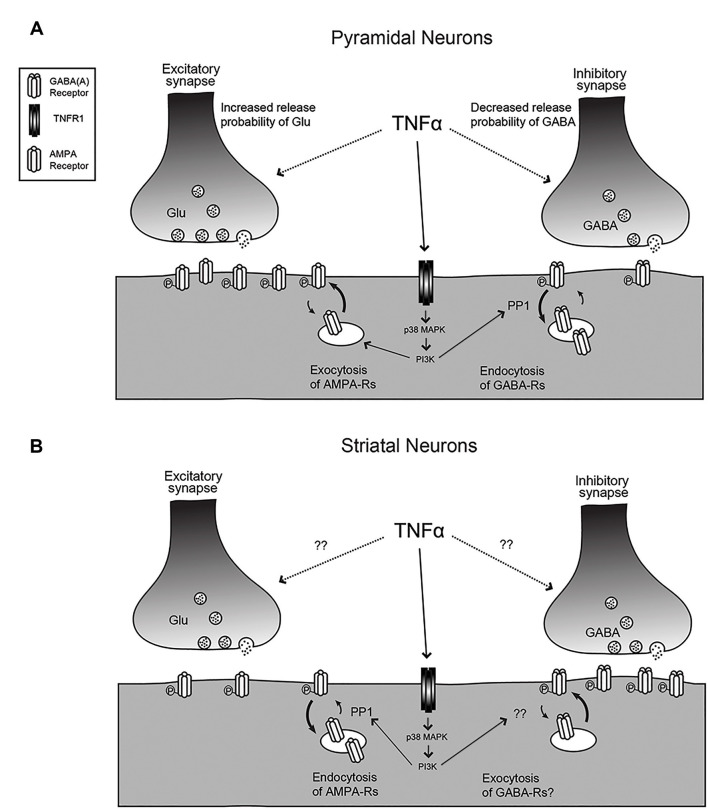
The effects of tumor necrosis factor (TNF) on synaptic function. **(A)** For hippocampal or cortical pyramidal neurons, TNF treatment leads to an increase in release probability and an increase in AMPA receptor content at excitatory synapses but a decrease in release probability and decrease in GABA_A_ receptor content at inhibitory synapses. The mechanisms for the change in release probability are unknown but the post-synaptic receptor trafficking requires p38-MAP kinase and PI3 kinase and the receptor endocytosis is dependent on protein phosphatase 1 (PP1). **(B)** The response is reversed for medium spiny neurons (MSNs) in the striatum and neurons in the habenula. Here, TNF causes endocytosis of AMPA receptors and may cause exocytosis of GABA receptors. Changes in release probability have not been documented. Figure adapted from Pribiag and Stellwagen (2013).

TNF is capable of modulating both presynaptic and postsynaptic function in neurons (Figure 1A and Table 1). A key measure of the presynaptic function is the frequency of miniature postsynaptic currents, which are the post-synaptic response to the unitary release of neurotransmitters. The frequency of these currents is generally taken to be a reflection of the probability of release of transmitter from the presynapse, as well as the number of synapses on the cell, while changes in amplitude are generally assumed to be due to post-synaptic changes. It should be noted that there are several ways these assumptions can fail, but they hold true for most situations. Treatment of cultured hippocampal neurons with TNF increases miniature excitatory postsynaptic current (mEPSC) frequency in pyramidal neurons (Grassi et al., 1994; Beattie et al., 2002). This effect was observed during direct, short term application of TNF to individual neurons, but is more difficult to detect with longer-term treatments and cross-cell comparisons (e.g., Stellwagen et al., 2005; Stellwagen and Malenka, 2006). Whether the increase in release probability is temporary or whether it is lost in the noisiness of cross-cell comparisons is uncertain. One report suggests that the effect on release probability may not be direct, but rather through a mechanism involving the glial release of other factors such as ATP (Santello et al., 2011).

**Table 1:** Details of the effects of tumor necrosis factor (TNF) on synaptic function.

Preparation	TNFα treatment	Result	Reference
Rat hippocampal cultures	50–180 ng/ml, 2–5 min	↑ Glutamate release probability	Grassi et al. (1994)
	10–1,000 ng/ml, 15 min	↑ Glutamate release probability ↑ Surface AMPARs	Beattie et al. (2002)
	50–250 ng/ml, 45 min	↓ GABA release probability ↓ Surface GABARs ↓ GABAR current	Pribiag and Stellwagen (2013)
	100 ng/ml, 15–20 min	↑ Surface AMPARs ↑ AMPAR current ↓ Surface GABARs ↓ GABAR current	Stellwagen et al. (2005)
Mouse acute hippocampal slices	100 ng/ml, 15 min	↑ Surface AMPARs	Ogoshi et al. (2005)
Rat acute hippocampal slices	1,000 ng/ml, 2–3 h 100 ng/ml, 1–2 h	↑ AMPAR current ↓ GABAR current	Stellwagen et al. (2005) and Lewitus et al. (2014)
Rat acute striatal slices	100 ng/ml, 1–2 h	↓ AMPA/NMDA ratio ↓ Surface AMPARs	Lewitus et al. (2014, 2016)
Rat acute lateral habenula slices	100 ng/ml, 1 h	↓ AMPA/NMDA ratio	Valentinova et al. (2019)

The modulatory effects of TNF are not unique to excitatory synapses—miniature inhibitory synaptic current (mIPSC) frequency decreases with TNF treatment of hippocampal cultures (Pribiag and Stellwagen, 2013). Furthermore, the application of a soluble version of TNFR1, which serves to block TNF signaling by acting as a TNF sink, results in a decrease in the baseline mEPSC frequency, suggesting that ongoing TNF signaling is required to maintain normal synaptic function. This indicates that not only is TNF capable of modulating synaptic function in response to its administration, but also that its constitutive secretion is responsible for maintaining synapses in their baseline state. Taken together, these effects are all consistent with an overall outcome of increased synaptic transmission in the presence of TNF, suggesting an important role of TNF under non-pathological, non-inflammatory conditions in the CNS.

The most well-established mechanism by which TNF modulates synapses is through the post-synaptic trafficking of neurotransmitter receptors. Excitatory neurotransmission is mainly accomplished through the activation of α-amino-3-hydroxy-5-methyl-4-isoxazolepropionic acid-type glutamate receptors (AMPARs), and their abundance at the synapse largely determines the neuronal response to a given stimulus. They are therefore a frequent point of regulation for the expression of synaptic plasticity (Malinow and Malenka, 2002).

Early studies focused on the effects of exogenous TNF administration on mature cultured hippocampal and cortical neurons. Treatment of dissociated hippocampal cultures with TNF results in a rapid (within 10–15 min) and large-scale trafficking of AMPARs (doubling) to the surface of pyramidal neurons, as determined by immunocytochemistry (Beattie et al., 2002; Ogoshi et al., 2005; Stellwagen et al., 2005). These newly-inserted receptors colocalize with synaptic markers, indicating that they can contribute to synaptic function (Beattie et al., 2002). It is also important to note the potential role of TNF in setting basal AMPAR levels. Application of a soluble version of TNFR1 resulted in the reduction of surface AMPAR staining to below baseline (Beattie et al., 2002), again suggesting that TNF is important for continual maintenance of synaptic components. Also, cultured cortical neurons prepared from TNFR1 knockout animals have fewer surface GluA1 clusters (He et al., 2012), confirming a role for TNF in maintaining AMPAR levels.

The synaptic effects of TNF were also more directly tested by electrophysiology on both cultured neurons and the more intact preparation of acute hippocampal slices. The amplitude of miniature postsynaptic currents are the neuronal response to the unitary release of neurotransmitters, and as such, it is taken to be reflective of the receptor content of the postsynaptic cell. Administration of TNF to both dissociated neuronal cultures and acute slices resulted in an increase in mEPSC amplitude on pyramidal neurons (Stellwagen et al., 2005), which is consistent with immunocytochemistry data indicating that TNF strengthens synapses. It is interesting to note that longer-term exposure to TNF can lead to different results—24 h treatment led to a modest decrease in whole-cell AMPA-induced currents (Furukawa and Mattson, 1998), indicating that time course may play a role in the biological outcome of TNF exposure.

It is also important to consider the subunit composition of AMPARs, as it is critical to their biological function. AMPARs are assembled as tetramers of the GluA1–GluA4 subunits (Wisden and Seeburg, 1993). In general, they are found as heteromers of either GluA1 and GluA2, or heteromers of GluA2 and GluA3, but can be found as GluA1 homomers (Wenthold et al., 1996; Shi et al., 2001). The presence of GluA2 in receptor complexes is critical: it is the subunit that confers calcium impermeability to the AMPAR complex (Burnashev et al., 1992). The biological consequences of calcium permeability are wide-reaching due to the importance of calcium to many synaptic processes. It is critical to multiple forms of plasticity (Zucker, 1999), and is part of the cascade of excitotoxic cell death which is characteristic of numerous neurological pathologies (Choi, 1992; Dong et al., 2009). It is then particularly intriguing that several groups have reported that the AMPARs trafficked to the cell surface in response to TNF treatment are permeable to calcium (Ogoshi et al., 2005; Stellwagen et al., 2005) because of the potential implications for neurological disease, which often involve neuroinflammation. It has also been reported that after initial, rapid exocytosis of GluA2-lacking receptors within minutes, AMPARs are slowly switched to GluA2-containing surface receptors (Leonoudakis et al., 2008) with longer treatment, suggesting that the outcome of TNF application is dependent on the time course of application, and responses may occur in more than one phase.

TNF can also modulate inhibitory neurotransmitter receptors. γ-aminobutyric acid receptors (GABARs) are the main mediators of fast inhibitory transmission in the brain (Jacob et al., 2008) and are critical to the dynamics of neural circuits. An early study in hippocampal culture and acute hippocampal slices shows that TNF treatment leads to both a decrease in surface GABAR staining, as well as a decrease in mIPSC amplitude, consistent with an overall decrease in inhibitory neurotransmission (Stellwagen et al., 2005). A subsequent report determined that the mechanism of TNF-induced GABAR regulation is through p38 MAPK, PI3K, and protein phosphatase 1 (PP1), leading to the dephosphorylation of the GABARs and their endocytosis from the cell surface (Pribiag and Stellwagen, 2013). Taken together, the overall effect of TNF-induced receptor trafficking—increased surface AMPARs and decreased surface GABARs—is to increase the strength of synapses. Because exogenous administration is capable of rapidly modulating both excitatory and inhibitory synapses, TNF emerges as a potentially critical regulator of circuit excitability.

## TNF Effects on Striatal Neurons

In addition to this detailed work on the effects of TNF on the glutamatergic neurons of the hippocampus and cortex, its function has also been characterized on the inhibitory medium spiny neurons (MSNs) of the striatum (Figure 1B). In experiments where acute striatal slices were treated with TNF, there was a decrease in excitatory synaptic strength in corticostriatal synapses as measured by electrophysiology, as well as a decrease in surface AMPARs measured biochemically (Lewitus et al., 2014). These changes are more prominent on the direct pathway MSNs than on indirect pathway neurons (Lewitus et al., 2016). It is intriguing that in this context, the AMPARs that are trafficked are GluA2-lacking receptors, the same subtype that is trafficked in response to TNF in the hippocampus, although in the opposite direction. While this initially appears contradictory, the result of a decrease in excitatory synaptic strength in the striatum is a decrease in its inhibitory output through MSNs. Therefore, the overall effect of TNF administration is increasing the strength of neural circuits, which is consistent with the overall effect in the hippocampus and cortex.

## HSP in Dissociated Culture

The exogenous application of TNF has clear effects on synapses, so it is, critical to consider the biological conditions that lead to TNF release in the CNS. Examining the role of TNF in various forms of synaptic plasticity, therefore, gives context to the effects on neurotransmitter receptor trafficking observed by TNF administration.

TNF is critical to the process of scaling up excitatory synaptic strength in response to prolonged activity blockade (Stellwagen and Malenka, 2006). Depriving dissociated hippocampal cultures of activity for 48 h using tetrodotoxin (TTX) to prevent action potential generation by blocking sodium channels results in an increase in surface AMPARs and a decrease in surface GABARs. This modulation of surface receptors gives rise to the expected electrophysiological changes: mEPSC amplitude increases, while mIPSC amplitude decreases, giving an overall increase in synaptic strength. Synaptic changes are accompanied by an increase in TNF in the cell culture medium, suggesting that it could be involved in the response to activity deprivation. This is supported by experiments showing that treatment of cultures with a soluble TNFR1, which blocks TNF signaling, during activity blockade prevents the upscaling of synaptic strength. Furthermore, TNF KO animals lack HSP in response to activity deprivation, supporting its involvement in synaptic strengthening. Altogether, this is clear evidence that TNF is required for synaptic upscaling. It is important to note, however, that TNF does not appear to be required for the downscaling of synapses in response to activity elevation (Stellwagen and Malenka, 2006).

Interestingly, there is a report suggesting that the TNF requirement in HSP is time-dependent (Steinmetz and Turrigiano, 2010). TNF may be dispensable for early (<6 h) stages of HSP, but that its prolonged blockade with a soluble TNFR1 does prevent late (24 h) stages of HSP, which is not necessarily inconsistent with previous reports characterizing TNF involvement in the response to 48 h of activity blockade. Rather, it implies that there are two stages to the process of scaling up synapses. Further experimentation will be required to determine the distinctions between early and late phase HSP as it relates to TNF.

The precise subunit composition of the AMPARs trafficked during HSP has not been completely characterized. However, increases in surface GluA1 staining were observed following activity deprivation (Stellwagen and Malenka, 2006). Together with previous evidence in the same culture system showing that TNF treatment resulted in exocytosis of GluA2-lacking AMPARs (Stellwagen et al., 2005), it seems likely that the same type of AMPARs would be trafficked in this form of HSP. Furthermore, other reports of TTX-induced homeostatic plasticity are generally supportive of this, showing increased levels of GluA2-lacking AMPARs after activity deprivation (Thiagarajan et al., 2005; Sutton et al., 2006; Aoto et al., 2008; Hou et al., 2008). Additionally, there is a report suggesting that phosphorylation of GluA1 is required for synaptic scaling (Kim and Ziff, 2014). Some reports show that GluA2 is required for TTX-induced scaling using GluA2 knockdown cortical cultures (Gainey et al., 2009) and organotypic hippocampal slice cultures (Ancona Esselmann et al., 2017). On the other hand, a study was also performed using knockout cultures for GluA1, GluA2, and GluA3 indicating that there is no subunit requirement for TTX-induced upscaling (Altimimi and Stellwagen, 2013), perhaps as a result of compensation by alternate compositions of AMPARs in the absence of a given subunit.

Understanding the source of TNF during HSP gives valuable insight into the mechanics of the process. Early studies in culture indicated that glia produce TNF basally, and that conditioned media from glial cultures was able to induce exocytosis of AMPARs neurons (Beattie et al., 2002), but it was not clear whether this was the mechanism at play during HSP. Using Banker cultures to plate neurons onto a feeder layer of glia that is physically separate, a genetic approach allowed for precise characterization of the roles of individual cell types in TNF secretion. Wild type neurons cultured with TNF KO glia were unable to express HPS whereas TNF KO neurons cultured with wild type glia behaved similarly to controls suggesting that glial TNF mediates HSP (Stellwagen and Malenka, 2006).

While implicating glia, this work did not identify the subtype involved. Within the central nervous system, TNF is largely produced by glia, including both astrocytes and microglia. TNF is occasionally seen (both at the RNA and protein level) in neurons, but typically only in pathological conditions. Which cells secrete the low level of TNF regulating HSP is currently unclear. *In vivo*, varying manipulations result in TNF production from astrocytes (Duseja et al., 2015) and microglia (Lewitus et al., 2016). During HSP, astrocytes are the best positioned to monitor the activity of synapses and are the likely source of HSP-mediating TNF, but this remains to be determined.

For many years, glia were assumed to merely provide physical and trophic support for neuronal function. The finding that glial TNF is required for HSP lends weight to the more recent observation that glia are capable of being active players at the synapse, shaping properties of neurotransmission through the secretion of modulatory factors.

## HSP in Entorhino-Hippocampal Slice Cultures

While dissociated culture is a valuable tool for the dissection of neural function, it is important to verify the biological relevance of the information gleaned from them. As such, performing experiments in more intact systems is necessary to examine whether findings in dissociated culture hold true outside of that system. In that vein, HSP has been studied in entorhino-hippocampal slice cultures.

The entorhinal cortex is the major input and output structure for the hippocampus and is often thought of as a gateway between the hippocampus and cortex. Projections from the entorhinal cortex to the dentate gyrus termed the perforant path, have been studied extensively in terms of both structure and plasticity for many years (Bliss and Lomo, 1973; Douglas and Goddard, 1975; Witter, 2007). These connections can be preserved in a slice culture system, allowing for the examination of a physiologically relevant neural circuit in the context of HSP.

Entorhino-hippocampal slice cultures also allow for a more physiological activity manipulation than bath application of TTX. Entorhinal denervation by lesioning the inputs to the dentate provides a paradigm in which synapses in the dentate can be studied in terms of their response to a decrease in excitatory input. When this type of lesion is performed, it results in a homeostatic increase in mEPSC amplitudes in the dentate which reaches its maximum 3–4 days post-lesion (Vlachos et al., 2012, 2013), similar to the effects of TTX treatment on dissociated cultures. Further addition of TTX to denervated slice cultures did not lead to an additional increase in synaptic strength, suggesting that a common pathway underlies the response to both manipulations.

TNF was also required for this form of HSP. Slice cultures either made from TNF KO animals, or slice cultures treated with soluble TNFR to block signaling lacked the late-stage synaptic strengthening at 3–4 days post-lesion (Becker et al., 2013). Furthermore, TNF is likely secreted by glia in this context, similar to early HSP experiments. Using *in situ* hybridization in concert with immunofluorescent labeling of astrocytes, the authors show an increase in TNF mRNA in astrocytes after denervation. Though this does not exclude a contribution of TNF from other cell types, it does suggest that astrocytes are capable of supplying TNF during denervation-induced HSP, though further experimentation is necessary to ascertain whether astrocytic TNF is a requirement.

It is, however, important to note that not all components of HSP are recapitulated in the slice culture denervation model. Recently, a study showed that while increased mEPSC amplitude in dentate granule cells is observed in slice culture, there is no concomitant decrease in mIPSC amplitude (Lenz et al., 2019) that is characteristic of HSP in dissociated neuron-glial cultures (Turrigiano et al., 1998; Kilman et al., 2002; Stellwagen and Malenka, 2006). The use of *in vitro* models has utility due to the ease of performing manipulations, but must be validated *in vivo*.

## TNF in Meta-Plasticity

There is also evidence that TNF is involved in forms of plasticity other than HSP. While TNF regulates AMPA receptor trafficking, it is not required for either LTP or LTD (Stellwagen and Malenka, 2006). However, TNF may be capable of altering the threshold of induction for these Hebbian forms of plasticity in a process called meta-plasticity. Prior TNF treatment can inhibit or reduce subsequent hippocampal LTP in various circumstances (Tancredi et al., 1992; Cunningham et al., 1996; Butler et al., 2004; Pickering et al., 2005), often at lower doses and shorter applications than for TNF-mediated receptor trafficking. Recent work has clarified these findings, determining that TNF is capable of inducing meta-plasticity (Hulme et al., 2012; Singh et al., 2019), where prior activity reduces the induction of LTP but increases the induction of LTD (Hulme et al., 2012). The mechanism for this meta-plastic shift is uncertain but may be distinct from HSP. The relationship between meta-plasticity and HSP is also currently unclear—both can provide stability to neural networks, and may represent aspects of a larger, integrative negative feedback system. Importantly, many of the *in vivo* functions of TNF discussed below induce synaptic changes that rely on sustained TNF signaling, and so are more likely due to its role in HSP rather than meta-plasticity. But further work will need to clarify the situation for any particular *in vivo* function for TNF.

## Monocular Deprivation-Induced Plasticity

The visual system has also offered insight into the role of TNF-dependent plasticity in intact animals. During early development, the visual system is highly plastic at a time referred to as the critical period (Hubel and Wiesel, 1970). If one eye is deprived of input by suturing it shut—an experimental paradigm termed monocular deprivation (MD)—several stages of plasticity are engaged in the binocular zone of the visual cortex, which altogether are referred to as ocular dominance plasticity. First, evoked responses to visual stimulation of the closed eye are rapidly decreased, which is followed by an increase in responses to stimulation of the open eye in the binocular cortex (Frenkel and Bear, 2004). The temporal separation of these two events suggests that they are distinct processes that are likely mechanistically divergent.

TNF is required for the open eye potentiation phase of plasticity after MD (Kaneko et al., 2008). Using both single-unit recordings and intrinsic optical imaging techniques (where neural activity is assessed by changes in reflectance of the brain surface), Kaneko et al. (2008) show that TNF knockout animals lack this increase. Furthermore, cortical infusion of a soluble TNFR to block TNF signaling during deprivation phenocopies the result. This recapitulates the overall features of HSP: a homeostatic response to a decrease in synaptic input requiring TNF.

## Whisker Deprivation

TNF has also been implicated in homeostatic plasticity in the somatosensory cortex. Trimming or plucking rodent whiskers to decrease input into the barrel cortex results in a rapid decrease in response to stimulation of deprived whiskers, followed by a slower increase in responses to neighboring spared whiskers when performed in a critical period of development (Glazewski and Fox, 1996), echoing plasticity in the visual cortex after MD. In the barrel cortex, however, the expression of plasticity has been studied in terms of cell type as well: regular spiking (RS) and intrinsic bursting (IB) pyramidal neurons behave differently (Greenhill et al., 2015). In layer 5 of the barrel cortex, unilateral whisker trimming deprivation leads to an initial depression of deprived whisker responses, followed by a slower potentiation in both RS and IB cells above original baseline levels. Critically, the potentiation is multiplicative, indicating that the plasticity is indeed HSP. This represents yet another instance where HSP occurs *in vivo*, mirroring experiments conducted in culture. However, if only one row of whiskers is trimmed, there is an initial decrease in deprived whisker responses only in RS cells, followed by a slower potentiation in both cell types that is not multiplicative, suggesting a more complex mechanism is at play when deprivation is not complete, perhaps involving multiple modalities of plasticity in addition to HSP. The authors also tested the TNF dependence using knockout animals of barrel cortex plasticity and found that the recovery from the initial potentiation in both cell types requires TNF. However, potentiation above baseline levels was only dependent on TNF in RS cells.

## Hearing Loss

TNF-dependent HSP has a clear role in two different sensory cortices, so it is interesting to speculate that HSP is a general response to sensory deprivation. Indeed, this has been examined in the auditory system using a model of conductive hearing loss (CHL) in adult mice (Teichert et al., 2017). In the primary auditory cortex, there is an initial decrease in responsiveness to auditory stimuli. After 3 days of CHL, there is a multiplicative increase in synaptic strength in the cortex, indicating it is the result of HSP. Additionally, recovery of responses to intense stimuli is impaired in TNF knockout animals, further implicating TNF in that potentiation.

Therefore, TNF-mediated HSP underlies the response to sensory deprivation in three different modalities, suggesting that it may be a general response to a decrease in sensory input. Furthermore, HSP is part of the response to changes in sensory experience in an intact animal, emphasizing that it is an important mechanism with biological relevance outside of the culture dish. It should be noted, however, that there are some differences in the expression of plasticity between the modalities. For example, ocular dominance plasticity after MD does not require TNF in an adult animal (Ranson et al., 2012). On the other hand, the hearing loss-induced HSP experiments described by Teichert et al. (2017) above were conducted in adult mice and required TNF for some components of the homeostatic response. While the existence of experience-dependent plasticity requiring TNF appears to be common to the modalities, the rules governing its expression may differ between cortical areas.

## Behavioral Response to Antidepressants

TNF function in sensory cortex plasticity is consistent with a role in the response to decreased sensory input, which is an intuitive extension of the role of TNF in activity-induced HSP. TNF, however, seems to play a role in the behavioral response to antidepressants as well, which may point to a more complex function in that system.

Plasma levels of pro-inflammatory cytokines including TNF are elevated in patients with major depressive disorder (MDD; Dowlati et al., 2010), and polymorphisms in the TNF gene that modulate its expression may contribute to susceptibility to MDD (Cerri et al., 2009). At the molecular level, antidepressant treatment of rats results in increased glutamate receptor expression (Barbon et al., 2011) and synaptic localization (Ampuero et al., 2010). Therefore, the involvement of TNF in the mechanism of antidepressant action would be intriguing because it is established that TNF can modulate glutamate receptors.

This is indeed the case, as described in a report using TNF deficient mice in an animal model of depressive behavior (Duseja et al., 2015). Using two tests of depressive-type behavior, the forced swim test (FST) and tail suspension test (TST), the authors show that TNF is required for the amelioration of depressive phenotypes, a standard test for the efficacy of antidepressants. While wild type animals showed a decrease in immobility in both of these tests after administration of two different antidepressants, fluoxetine and desipramine, TNF KO animals showed no response until a much higher dose of antidepressant was used. Furthermore, the phenotype was recapitulated in GFAP-Cre, TNF-flox animals, which only lack TNF in astrocytes, suggesting that this cell type is responsible for the effect of TNF on antidepressant action. This is particularly intriguing, as the TNF released during HSP in entrohino-hippocampal slice cultures is also likely of astrocytic origin (Becker et al., 2013), raising the possibility that a similar homeostatic mechanism is at play during antidepressant administration. Furthermore, the fact that antidepressant administration does not have an immediate effect on depressive behaviors, but rather takes several weeks to reach efficacy, is consistent with a homeostatic process in its mechanism of action.

## TNF Effects on Striatal Function

The striatum, which functions to process information in the basal ganglia, receives input from the cortex, brainstem, and thalamus and integrates those inputs to facilitate voluntary movement as well as integrate cognitive and motivational information. It is fundamentally different from the hippocampus and cortex, which are comprised of large numbers of excitatory neurons, in that it is made up of almost exclusively of inhibitory MSNs that form its only output (Gerfen and Wilson, 1996). As noted above, the TNF response of MSNs is inverted from that of pyramidal neurons (Lewitus et al., 2014). However, TNF still appears to function in an adaptive or homeostatic context in this structure.

## Adaptive Response to Striatal Dysfunction

Chronic administration of antipsychotic drugs such as haloperidol, which block D2 dopamine receptors, can result in extrapyramidal symptoms such as tardive dyskinesia (involuntary face movements) as a result of dysregulation of the striatal circuit responsible for movement. These symptoms are accompanied by both increased TNF levels as well as increased AMPA binding, raising the possibility of HSP-type mechanisms contributing to this pathology (Schmitt et al., 2003; Bishnoi et al., 2008). Blocking TNF in animals treated with haloperidol by using a dominant-negative form of the cytokine results in more frequent involuntary movements, indicating that when present, TNF functions to limit the effects of chronic haloperidol on the corticostriatal circuit (Lewitus et al., 2014). The authors of that study further show that this is through the endocytosis of GluA2-lacking AMPARs, which are trafficked in response to TNF. Altogether, this indicates that TNF is critical to a homeostatic process that serves to counter corticostriatal circuit perturbations.

## Behavioral Sensitization to Cocaine

The administration of drugs of abuse to animals leads to an increase in striatal dopamine, which is accompanied by changes in glutamatergic transmission in the nucleus accumbens (NAc) of the striatum. More specifically, repeated administration of cocaine to rodents results in an initial decrease in AMPA/NMDA ratio in the NAc 24 h after the last injection, followed by a gradual increase in AMPA/NMDA ratio during a period of abstinence after that (Kourrich et al., 2007). Given that TNF can modulate AMPAR content in the striatum (Lewitus et al., 2014), it became an interesting possibility that TNF could be playing a role in circuit dynamics in a model of cocaine addiction.

A behavioral readout of responses to cocaine administration is the extent of sensitization to cocaine exposure. When given repeatedly, cocaine causes an increasingly large locomotor response, termed behavioral sensitization, and its expression depends on AMPAR content in the NAc (Kalivas, 2009). In a study using a dominant-negative form of TNF, the authors find that when TNF is blocked, they observe both increased behavioral sensitization as well as an exaggerated potentiation of synapses onto D1-type MSNs with no initial depression, suggesting that TNF in this system serves to limit the effects of cocaine administration (Lewitus et al., 2016). Furthermore, the source of TNF in this model is microglia, as this result can be phenocopied by carrying out the same experiment in CX3CR1-Cre, TNF-flox mice which lack TNF only in microglial cells. Thus, TNF is placed within another adaptive pathway that serves to limit changes in striatal circuitry.

## Morphine Withdrawal

TNF appears to play a role in the response to other drugs of abuse in addition to cocaine. In morphine withdrawal models, TNF plays a role in synaptic adaptations after cessation of drug administration (Valentinova et al., 2019). These changes occur in the lateral habenula, an area that both processes aversive stimuli and regulates monoaminergic systems. TNF is only slightly elevated by morphine administration but increases dramatically during withdrawal. Valentinova et al. (2019) find that during withdrawal, there is a decrease in synaptic strength (as measured by AMPA/NMDA ratios) in the medial aspect of the lateral habenula, specifically in raphe-projecting neurons, which requires neuronal TNFR1 signaling. Further downstream, increased TNF signaling results in decreased sociability that is a hallmark of withdrawal symptoms. That excitatory neurons in this system have a TNF-mediated decrease in synaptic strength suggests that neurons cannot simply be divided into excitatory vs. inhibitory neurons to determine the direction of TNF-mediated changes and that the property of individual subtype of neurons (excitatory and inhibitory) must be directly tested. The work also suggests that while TNF reduces circuit changes in the NAc during drug administration, it may drive changes in other parts of the reward system, so effects across the whole circuit must be considered.

## Conclusions

TNF is well known to have pleiotropic effects, allowing it to coordinate many functions under different circumstances and conditions. Within the immune system, various cell types will respond in distinct ways to coordinate the inflammatory response. We suggest that TNF may play a similar pleiotropic role in regulating neuronal circuit function. It is clear that the effects of TNF on neurotransmission are neuronal subtype-specific, and that it can lead to several different outcomes at the level of synapses. However, the common thread is that these changes still appear to normalize circuit output in response to perturbing stimuli, which is consistent with TNF being a mediator of HSP. Thus, TNF-induced trafficking of neurotransmitter receptors in the CNS may be a general mechanism by which circuit homeostasis and function are maintained both *in vitro* and *in vivo*. Disrupting TNF signaling can, therefore, be a route to investigating the role of HSP *in vivo*. However, TNF-mediated changes can also be driving changes in circuit function, as seen during morphine withdrawal. TNF-mediated HSP could also become dysregulated under pathological conditions, leading to TNF driving maladaptive changes in circuit function. Whether TNF is acting in an adaptive or maladaptive manner must be assessed for individual circuits in response to particular situations.

## Author Contributions

RH and DS wrote the manuscript.

## Conflict of Interest

The authors declare that the research was conducted in the absence of any commercial or financial relationships that could be construed as a potential conflict of interest.
